# Research Progress on the Molecular Mechanism of Poultry Feather Follicle Development

**DOI:** 10.3390/cimb47090684

**Published:** 2025-08-25

**Authors:** Jiangxian Wang, Shiliang Zhu, Xia Xiong, Mohan Qiu, Zengrong Zhang, Chenming Hu, Li Yang, Han Peng, Xiaoyan Song, Jialei Chen, Bo Xia, Zhuxiang Xiong, Longhuan Du, Chunlin Yu, Chaowu Yang

**Affiliations:** Animal Genetic Breeding and Reproduction Key Laboratory of Sichuan Province, Sichuan Animal Science Academy, Chengdu 610066, China; wangjiangxian@scsaas.cn (J.W.); zhushiliang1994@163.com (S.Z.); xiongxia20120904@163.com (X.X.); mohan.qiu@163.com (M.Q.); zhangzengrong2004@163.com (Z.Z.); huchenming@126.com (C.H.); yangli_sasa@163.com (L.Y.); penghan0706@163.com (H.P.); babalasxy@163.com (X.S.); qiaoqiaowo110@163.com (J.C.); allanbobo777@163.com (B.X.); xiongzhuxiang@scsaas.cn (Z.X.); longhuan_du@scsaas.cn (L.D.)

**Keywords:** feather follicle development, poultry, molecular mechanism

## Abstract

The evolution of the chilled processing technology has precipitated the emergence of ice-fresh poultry meat as a significant sales channel. The aesthetic appearance of chicken carcasses has become increasingly important in the context of poultry ice-fresh sales, in conjunction with the comprehensive implementation of China’s policies for poultry. Feather follicle development is a significant factor in determining the aesthetic appearance of the carcass. Recent studies have focused on the molecular mechanisms associated with feather follicle development. The WNT, EGF, FGF, SHH, and BMP signalling pathways have been identified as the regulatory mechanisms involved in the development of feather follicles in various segments of poultry skin. However, the BMP signalling pathway, acting as an inhibitor, has been demonstrated to impede the regulatory processes governing feather follicle development via these signalling pathways. This review summarises the structure and overview of feathers and feather follicles, the research progress of signalling pathways that affect the development of poultry feather follicles, the research progress of poultry follicle traits, and the research progress of feather follicle development biotechnology. The present review focuses on summarising the molecular mechanisms that affect feather follicle development, and on providing a summary of the application of biotechnology in this field. It also offers ideas and theoretical references for the molecular mechanism of poultry feather follicle development.

## 1. Introduction: The Structure and Overview of Feather and Feather Follicles

### 1.1. The Structure and Overview of Feather

The first documented appearance of feathers on dinosaurs was during the Jurassic period, approximately 150–160 million years ago. A type of theropod dinosaur that possessed feathers survived the mass extinction and became the ancestor of birds [1]. Feathers represent a novel organ system that evolved from the exoskeleton of dinosaurs. Recent research in the field of developmental biology, coupled with the findings of numerous fossil discoveries, has provided substantial evidence to suggest that the evolution of feathers was driven by a series of novel morphogenetic events [2,3]. The feathers of poultry are believed to be an evolutionary descendant of scales [4].

#### 1.1.1. The Structure and Classification of Feathers

**Figure 1:**
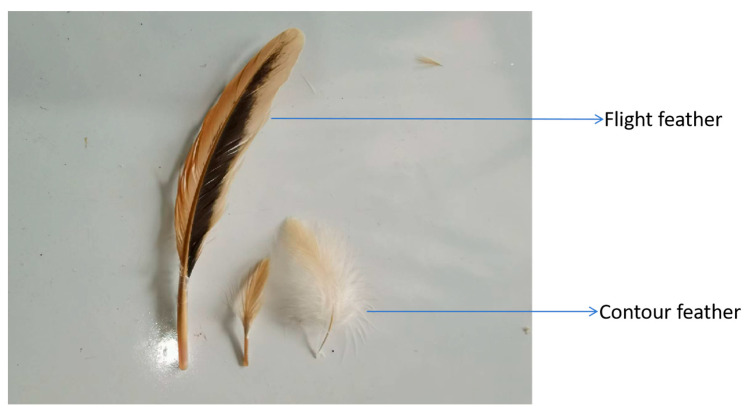
The classification of feathers.

Feathers are the keratinized products of epidermal cell proliferation in birds. The feathers of domestic poultry exhibit a wide range of branching structures [5]. The branches of feathers originate during the initial phase of feather development, comprising three distinct levels: from the shafts to the barbs, from the barbs to the rachises, and from the rachises to the barbules or hooklets. The geometry of the components of the abdominal feathers was characterised using scanning electron microscopy, microcomputed tomography and imaging techniques. The feather was found to be divided into three zones: an outer zone (bard shafts and barbules), an inner zone (bard shafts and barbules), and a transition zone [6]. It is evident that, based on the periods of their morphological development, feathers can be classified into three distinct categories: downy feathers, contour feathers, and flight feathers (symmetrical contour feathers on both sides and bilateral asymmetrical flight feathers) (Figure 1) [7,8]. The function of feathers is determined by their type, and they play a primary role in thermoregulation, attracting mates, communication, flight, and skin protection [7,9,10].

#### 1.1.2. The Development of Feathers

The development of feathers is a complex biological process that involves various types of feathers and multiple developmental stages [11]. The growth of feathers comprises three distinct stages: the initiation stage, the growth stage, and the stationary stage. These stages are characterised by a specific periodicity [12]. The growth process of feathers in an immature state occurs during the initiation stage and the growth stage, while the stationary stage represents the mature state of feathers. Throughout their lifespan, birds undergo continuous moulting and regeneration of their feathers. Feathers are capable of undergoing a process of regeneration through natural moulting or by manual removal. Following three to four cycles of feather growth and replacement, poultries develop adult feathers [13].

Feathers are primarily constituted of the soft keratinous layer material that is formed by the α and β keratin gene family [14]. As posited by Ng et al., the keratin located on chromosome 2 of poultry’s has the potential to exert a substantial influence on the development of hard feather structures. In contrast, the keratin situated on chromosome 25 has been demonstrated to play a pivotal role in the formation of soft feather structures [15]. The development process of feathers is initiated by the interaction between epithelial cells and mesenchymal cells, usually involving a series of dynamic cellular processes [16]. The control of these dynamic cellular processes is influenced by several important factors, including growth factors and their receptors, cell adhesion molecules and their ligands, signal transduction molecules and transcription factors [17,18,19,20,21].

### 1.2. The Structure and Overview of Feather Follicles

The growth and development of feathers is subject to the regulation of feather follicles. Follicle size not only determines feather growth-rate by mass, but also directly the structural design (shape, number of barbs, etc.) of a feather [22]. It is evident that at differing physiological stages, a variety of colours, shapes and functions of feathers can be produced [13,23]. The fundamental structure of avian follicles bears a resemblance to that of mammalian follicles, yet the structural and developmental intricacies of avian follicles surpass those of mammalian counterparts [12,24]. The follicles are primarily composed of follicle sheath (outer root sheath), feather sheath (inner root sheath), pulp, feather bard ridges, collar bulge and so on.

The formation of feather follicles is a unique occurrence in the life of a poultry, taking place exclusively during the embryonic stage [25]. The formation of feather follicles is a result of the interaction between epithelial and mesenchymal cells, and these follicles are fundamental to the growth and development of poultry feathers [8]. The morphology of the feather follicles varies among different chicken breeds, different parts of the same breed of chicken, and at different developmental stages.

On day 10 of the embryo stage, the rapid proliferation of mesenchymal cells commences, thereby forming the dermis layer within the developing feather follicle wall. This process is typically concluded by days 11 to 12 of the embryo stage. As the columnar cells on the dermal surface accumulate, the feather papilla is formed, thereby providing nutrients for feather growth. During the embryo stage of goose development, spanning the 13 to 14 days, the feather papilla undergoes thickening, leading to the formation of the feather primordium and the epithelial layer. The epidermis undergoes further expansion to form the feather bud, which then undergoes further invagination to form the primary feather follicle. The formation of secondary feather follicles occurs on the 18th day of the embryo stage of the goose [26,27].

During days 7 to 9 of embryonic stage, the chicken embryo develops an epidermal basal plate above the dermal condensation of the skin. The skin becomes elevated, and the feather primordia commence differentiation on day 9 of the embryonic stage. Feather buds begin to emerge from days 10 to 11 of the embryonic stage, the anterior and posterior extremities of the feather buds undergo asymmetric elongation, the formation of the feather follicle wall or primary feather follicle is initiated by the invagination of the epidermis, a process that is facilitated by the feather primordia. From days 13 to 14 of the embryonic stage, the formation of the feather follicle cavity occurs. Mesenchymal cells undergo a process of differentiation, ultimately forming the feather papilla. From days 15 to 16 of the embryonic stage, the formation of feather buds, otherwise known as secondary feather follicles, occurs. The growth and development of the primary feather follicle and the secondary feather follicle are not mutually exclusive [24,28].

It has been proven in ducks that cell proliferation occurs in the epithelium on day 11 of the embryonic stage, resulting in the formation of feather buds. On day 15 of the embryonic stage, the primary feather follicles form, and the newly formed feather follicles are filled with the feather sheaths. On day 20 of the embryonic stage, the feather follicles and the feather sheaths are closely connected, forming a single layer, and the feathers completely cover the entire body [29,30]. Therefore, different species of poultry have different feather follicle development patterns (Table 1).

## 2. Research Progress on the Effects of the Feather Follicle Signalling Pathway

The development of feather follicles is initiated by the interaction between epidermal and dermal fibroblasts during the embryonic stage. This process can be categorised into three phases: the growth phase, the degeneration phase, and the quiescent phase [31,32]. A plethora of signalling pathways have been identified as being involved in the morphogenesis of feather follicles (Table 2), including the Wnt (wingless-type MMTV integration site family members), EGF (epidermal growth factor), FGF (fibroblast growth factor), SHH (sonic hedgehog signalling pathway), BMP (bone morphogenetic protein) signalling pathway and so on [33,34].

### 2.1. Wnt Signalling Pathway

**Figure 2:**
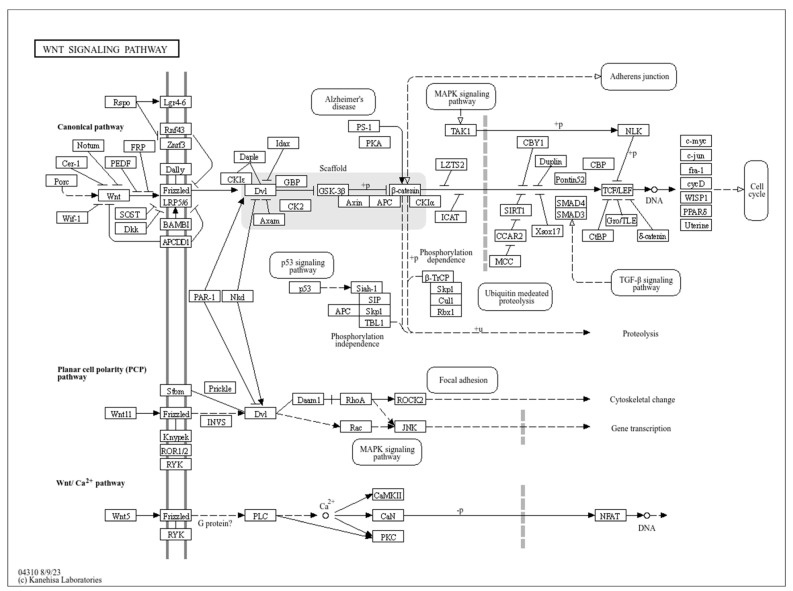
The canonical and noncanonical Wnt signalling pathways.

The Wnt family has been identified as comprising over 19 family members. Depending on the ligands and downstream factors, the Wnt signalling pathway can be broadly classified into the canonical and noncanonical pathways (Figure 2) [56]. The interaction between epidermal and dermal mesenchymal cells is a necessary condition for the morphogenesis of feather follicles [4]. The Wnt signalling pathway is the first signalling pathway to initiate the development of feather follicles by thickening the epithelial cells to form a substrate [35], which is a component of the biological process of embryonic development [36]. The dynamic changes in the Wnt signalling pathway have been demonstrated to induce the formation of feather follicles at different stages. The canonical Wnt signalling pathway is generally divided into two distinct regulatory processes: the upstream regulation of Wnt ligands and the downstream regulation of β-catenin during feather follicle development [37]. The *WNT7A* and *WNT9B* genes, via the Wnt signalling pathway, have been demonstrated to trigger epithelial thickening and the formation of feather follicle primordia, thereby exerting a significant influence on feather follicle development [38]. The core pathway for feather development is known to be Wnt/β-catenin, and this is subject to regulation at multiple levels by *FOXO3* (transcriptional layer) [39], miR-140-y (epigenetic layer) [40], and Met/PGAM5 (metabolic layer) [41]. It has been established that the Wnt/β-catenin signalling pathway exerts a pivotal regulatory influence on the initiation and spatial patterning of feather follicles. Ectopic Wnt activation has been demonstrated to induce de novo follicle formation, while the inhibition of this process has been shown to disrupt the establishment of primordial follicle arrays [57]. It is imperative to acknowledge the dose-dependent nature of these effects. Moderate Wnt expression has been observed to enhance follicle density and branching complexity; however, supraphysiological activation has been shown to result in fused follicles and cystic structures [58,59]. A regional genome editing strategy was developed by the injection of adenoviral CRISPR/Cas9 into specific sites of quail embryos (cervical flexure at HH13–15; limb bud at HH22–24). In post-hatch quail, the presence of grey feathers was exclusively observed at injection sites (i.e., the upper back or wing tips), thereby replicating the whole-body pigmentation patterns characteristic of *MLPH* knockout pigs [60]. The creation of homozygous *MLPH* knockout (HO) chickens was achieved through the utilisation of CRISPR/Cas9, which resulted in the introduction of a 1 bp deletion in the Rab-binding domain (RBD), leading to premature termination. It was observed that the HO chicks exhibited grey feathers at hatch, which is indicative of the anticipated *MLPH* phenotype. By five months of age, HO chickens exhibited symptoms of feather deterioration and loss (alopecia-like symptoms) on the wings and backs, unrelated to sex [61]. Consequently, the Wnt signalling pathway is regarded as playing a pivotal role in the morphogenesis, growth and development, distribution, as well as the growth and regeneration of feather follicles.

### 2.2. SHH Signalling Pathway

The Shh signalling pathway is one of the complex signal transduction mechanisms that control the developmental processes of multicellular organisms [62]. Shh is a secreted protein belonging to the highly conserved Hh (hedgehog) family. The gene responsible for encoding the Hh protein is referred to as SHH. The SHH, BMP, and Wnt/β-catenin signalling pathways collectively regulate the proliferation and differentiation of feather follicle cells. In poultry, the Shh signalling pathway plays a crucial role in the formation of feather follicles, from initiating skin coagulation to the formation of feather filaments [42]. Inhibition of the Shh signal has been demonstrated to result in reduced dermal coagulation, accompanied by spatial expansion and increased gene expression levels of BMP and WNT family genes [43]. Shh cooperates with the Wnt signalling pathway to upregulate the expression of Connexin-43, trigger Ca^2+^ channels, coordinate interstitial cell movement, and enhance feather bud elongation [44]. The SHH pathway has been shown to regulate feather branching through the reaction-diffusion model (SHH-BMP2), and it is the changes in its activity that appear to drive the evolution of feather morphology [45].

### 2.3. BMP Signalling Pathway

The BMP family has been identified as comprising more than 20 members, and it is subdivided into seven subgroups based on sequence homology and biological functions. The BMP then interacts with its receptors to form a complex, which in turn regulates the expression of downstream genes through the canonical Smad and noncanonical Smad signalling pathways [63,64]. The BMP signalling pathway plays a pivotal role in regulating a broad spectrum of biological functions across diverse cell types and tissues during both embryonic develop-ment and the postnatal growth period. Research has demonstrated that the BMP signal-ling pathway exerts a pivotal function in regulating epidermal cell differentiation and apoptosis during development. Furthermore, it has been shown to play a crucial role in pivotal steps of feather follicle development, including the initiation, cell fate determination and cell lineage differentiation. During the growth phase of feather follicles, the BMP signalling pathway plays a pivotal role in initiating growth and regulating feather follicle ageing driven by cell apoptosis [46]. The prevailing opinion amongst researchers is that the BMP signalling pathway exerts an inhibitory influence on feather follicle morphogenesis. An increase in BMPRIA or BMPRIB expression has been demonstrated to inhibit feather formation and increase the expression of *MSX1*, *MSX2* and *FGFR2* in the maxillary mesenchyme [47]. Furthermore, it has been demonstrated that *BMP4* over-expression can inhibit feather formation [48]. Research has identified that *BMP2* and *BMP7*, which are expressed in both the epidermis and dermis, exert opposing effects on cell condensation and feather patterns via the reaction-diffusion system [49]. BMP also inhibits multiple signalling pathways, including Wnt, Eda and FGF, in order to control the formation of feather follicles [47,48,49,50].

### 2.4. EGF Signalling Pathway

The epidermal growth factor (EGF) family consists of up to 13 members. The principal members implicated in the process of repairing corneal epithelial injury are epidermal growth factor (EGF), transforming growth factor-α (TGF-α) and heparin-binding EGF-like growth factor (HB-EGF) [65,66]. The EGF receptor belongs to the receptor tyrosine kinase family and plays a role in regulating the proliferation and differentiation of various cell types. The role of EGF signalling in establishing identity between buds is positive. The expression of both EGF and the active form of the EGF receptor (EGFR) has been observed in the feather bud area. Exogenous EGF has been demonstrated to stimulate epidermal proliferation and increase the expression of genes related to buds, while concomitantly causing a loss of feather bud gene expression and morphology. It has been demonstrated that EGF receptor signalling plays an active role in the promotion of feather bud development [51]. Research has demonstrated that transforming growth factor-beta-stimulated clone 22 (*TSC-22*) is dynamically expressed in the concentration zone of the feather bud and in numerous chicken embryo tissues. As previously demonstrated, the inhibitors of feather bud development, such as BMP-2/4, have been shown to suppress *TSC-22* expression during the process of feather formation in vivo. Noggin, a BMP inhibitor, has been demonstrated to promote *TSC-22* expression. Furthermore, EGF, TGF-α and fibroblast growth factors have been demonstrated to promote both feather bud development and *TSC-22* expression, and can induce ectopic feather buds between existing ones [52].

### 2.5. FGF Signalling Pathway

The FGF family comprises 18 secreted proteins and four tyrosine kinase receptors. These are divided into seven subfamilies based on sequence homology and biochemical properties. FGF signalling has been demonstrated to be associated with development, metabolism, and disease [67,68]. FGFs have been demonstrated to play a role in cell proliferation and morphogenesis during the earliest stages of embryonic development. It has been demonstrated that these cells can initiate the formation of feather patches and increase feather density in developing chicken skin [53]. FGF signalling has been demonstrated to act as an activator, driving the formation of feather primordia patterns during avian embryonic development [23]. The effects of signals and growth factors derived from platelet-derived mesenchymal stem cells on feather follicle growth and development have been demonstrated to be through cell proliferation, and it has been shown that the *FGF-7* gene prolongs the growth period of feather follicles [54]. *FGF20* has been demonstrated to induce Eda/Edar and Wnt/β-catenin signalling, functioning downstream [55].

## 3. Research Progress on the Characteristics of Poultry Feather Follicles

The primary feather follicle traits of poultry include number, density and diameter. The development of the feather follicles is completed during the embryonic stage, after which the number is fixed and will not change [25]. The number of feather follicles present varies between different species of bird. Research has demonstrated that this figure ranges of total follicles per bird from 1000 to over 10,000 [69,70]. The study of feather follicle density in poultry represents a pivotal research domain, with investigations focusing on the disparities among breeds and the influence of genetics. The study identified a total of 95 differentially expressed genes among the different feather follicle density groups. In contrast with the group exhibiting low feather follicle density, the group demonstrating high feather follicle density exhibited 56 genes that were found to be upregulated, as well as 39 genes that were downregulated. A total of 13 co-expressed gene modules were identified. A total of 103 core genes were identified as being non-essential to the function of the red module. The present study suggests the potential involvement of specific genes (namely, *FOXM1*, *GTSE1*, *MELK*, *CDK1*, *ECT2* and *NEK2*) in the development of feather follicle density in Wannan chickens [71]. The diameter of feather follicles is a significant indicator when evaluating the quality of poultry skin, as it directly impacts consumer perceptions of the appearance of chicken meat. Chen et al. conducted a study on the characteristics of feather follicles in the Wanxi White Goose breed, native to China, as well as on the polymorphisms of the *Wnt6* gene related to feather follicle development. The study revealed that the diameter of feather follicles on the chest and abdomen of the Wanxi White Goose was larger than on its back [72]. Ji et al. investigated the candidate genes responsible for chicken feather follicle traits, as well as conducting a gene co-expression network analysis of molecular pathways. This research discovered that the diameter of feather follicles on the legs of chickens was significantly larger than on the back. Furthermore, it was discovered that signalling path-ways related to feather follicle morphogenesis and development, such as Wnt, FGF, MAPK, SHH and BMP signalling pathway, played a key role in this co-expression network [73].

## 4. Research Progress in the Biotechnology of Poultry Feather Follicle

Transcriptomics research into the development of poultry feather follicles focuses primarily on changes in gene expression within feather follicle cells and their effect on the formation, development and periodic growth of feather follicles [74,75,76,77]. A transcriptomics study of poultry feather follicle development provides a new perspective from which to gain a deeper understanding of feather follicle biology. Moreover, it provides a scientific foundation for the enhancement and advancement of the poultry industry.

GWAS (Genome-Wide Association Study) research into the development of poultry feather follicles focuses primarily on identifying genetic markers that influence feather follicle traits. The aim is to provide a scientific basis for poultry breeding. GWAS is a method used to identify associations between complex traits and genetic variations. Adetula et al. conducted a genome-wide association study to identify genes associated with feather colouration. They found that the *RAI14* gene is a developmental regulatory gene encoding a protein containing numerous anchor protein repeat sequences, making it a candidate gene for feather colouration [78]. Guo et al. conducted a study using genome-wide association analysis to identify candidate genes for duck feather colouration, finding that the *MITF* and *EDNRB2* genes indirectly participate in the melanin formation pathway, thus establishing these as functional candidate genes for white and black feathers [79]. The application of GWAS research in the domain of avian feather follicle development is multifaceted. The application under discussion has two principal functions. Firstly, it assists in the identification and verification of genetic markers that affect feather follicle traits. Secondly, it promotes the progress of avian genetic breeding [80,81,82,83].

Genetics is the science that studies genes, mainly focusing on the direct alterations of DNA sequences and their effects on gene activity or function. These alterations include point mutations, deletions, insertions, and translocations. In contrast, epigenetics is the study of heritable changes in gene activity or function, but these changes do not involve any alterations to the DNA sequence itself [84]. Currently, more studies have been conducted on the mechanism of skin feather follicle development at the genome-wide DNA methylation level in mammals than in poultry [85,86,87]. Consequently, there is a paucity of research that is both comprehensive and in-depth into the molecular mechanisms underlying the characteristics of avian skin feather follicles. WGBS (Whole Genome Bisulfite Sequencing) is a high-throughput sequencing technology utilised for the study of DNA methylation, thereby facilitating comprehension of the epigenetic mechanisms that regulate gene expression. In the study of feather follicle development in poultry, WGBS has been shown to reveal the impact of DNA methylation on the feather follicle growth cycle and how to regulate the expression of feather follicle-related genes through changes in DNA methylation levels. This provides new insights into the regulation of feather follicle development.

## 5. Conclusions

The development of feather follicles in poultry occurs during the embryonic stage, and the number of feather follicles is determined at this point. The morphological development of feather follicles in poultry is subject to regulation by the Wnt, SHH, EGF, FGF and BMP signalling pathways. These signalling pathways have been demonstrated to be involved in the developmental process of feather follicles at different stages of feather follicle development. Nevertheless, it has been demonstrated that the BMP signalling pathway exerts a regulatory effect on other signalling pathways, thereby exerting a partial inhibitory effect on feather follicle development. Feather follicle development is a complex process that affects the characteristics of feather follicles and the appearance of the carcass. In order to provide a theoretical basis for new interventions to improve the appearance of poultry carcasses and to promote their commercial marketing, it is necessary to further investigate the molecular regulatory mechanisms of feather follicle characteristics by combining transcriptomics, genomics and epigenomics.

## Figures and Tables

**Table 1:** Growth and development stages of feather follicles in different poultry breeds.

Variety	Feather Formation	Primary Feather Follicle	Secondary Feather Follicle
Chicken	Embryonic days 10 to 11	Embryonic days 11 to 12	Embryonic days 15 to 16
Duck	Day 11 of the embryonic stage	Day 15 of the embryonic stage	Day 20 of the embryonic stage
Goose	Embryonic days 13 to 14	Day 14 of the embryonic stage	Day 18 of the embryonic stage

**Table 2:** This is basic information about the signalling pathways involved in feather follicle development.

Name	Family	Functions and Features	Regulation	Location of Action	References
Wnt signalling pathway	The Wnt family	The Wnt signalling pathway is the first signalling pathway to initiate the development of feather follicles	Positive	Epithelial cell	[35,36,37,38,39,40,41]
SHH signalling pathway	The Hh family	The process of skin coagulation is initiated, resulting in the formation of fine, feather-like structures	Positive	Feather	[42,43,44,45]
BMP signalling pathway	The BMP family	The BMP signalling pathway plays a crucial role in controlling epidermal cell differentia-tion and apoptosis during development, as well as in key steps of feather follicle development, such as initialisation, cell fate determination and cell lineage differentiation	Negative	Epithelial cell	[46,47,48,49,50]
EGF signalling pathway	The EGF family	The EGF signalling pathway plays a positive role in establishing identity between buds	Positive	Feather bud	[51,52]
FGF signalling pathway	The FGF family	The FGF signalling acts as an activator, driving the formation of feather primordia patterns during avian embryonic development	Positive	Feather primordia	[23,53,54,55]

## Data Availability

All data generated or analysed during this study are included in this published article.
